# Validation of the Life Metaphor Questionnaire Among Chinese College Students and Its Relationship with Meaning in Life: The Moderating Role of Depressive Symptoms

**DOI:** 10.3390/bs16101909

**Published:** 2026-10-15

**Authors:** Huan Lv, Xiaoyan Liang, Ruiming Wang, Keke Yu

**Affiliations:** 1 Mental Health Education and Counseling Center, Foshan University, Foshan 528225, China; lvhuanpsy@fosu.edu.cn; 2 Mental Health Education and Counseling Center, GuangDong Polytechnic, Foshan 528041, China; liangxiaoyan@gdpt.edu.cn; 3 Philosophy and Social Science Laboratory of Reading and Development in Children and Adolescents, Ministry of Education, Center for Studies of Psychological Application, School of Psychology, South China Normal University, Guangzhou 510631, China; wangrm@scnu.edu.cn

**Keywords:** life metaphor, meaning in life, depressive symptoms, college students

## Abstract

Life metaphors are cognitive tools for conceptualizing human existence and showing its essence, meaning, and characteristics. This study validated the Life Metaphor Questionnaire among Chinese college students and examined the relationship between life metaphors, meaning in life, and depressive symptoms. Study 1 (*n* = 1996) used item analysis, exploratory factor analyses (EFA), confirmatory factor analyses (CFA), and reliability analysis. The findings indicated that the 12-item questionnaire showed excellent internal consistency, and both the EFA and the CFA supported its three-factor structure, including fiction-related metaphors, uncertainty metaphors, and eudaimonic metaphors. Study 2 (*n* = 746) tested whether depressive symptoms moderated the relationship between life metaphors and meaning in life. The results showed all three life metaphor dimensions were positively correlated with meaning in life. Depressive symptoms were negatively correlated with fiction-related metaphors, eudaimonic metaphors, and meaning in life, but showed a very weak positive association with uncertainty metaphors. Depressive symptoms significantly moderated the associations of eudaimonic metaphors with both the presence of and the search for meaning; moderated the association of fiction-related metaphors with the presence of meaning, but not with the search for meaning; and did not moderate the associations of uncertainty metaphors with both the presence of and the search for meaning. The results not only advance understanding of how college students cognitively frame life meaning but also supply possible targets for future longitudinal or experimental research on meaning in life among college populations.

## 1. Introduction

The college years represent a critical transitional period in the life course. Empirical evidence indicates that many mental health difficulties among college students arise from a lack of meaning in life, which is a key vulnerability factor for psychological symptoms (de Vries et al., 2025; Hang et al., 2025). Consequently, improving meaning in life among college students has become a critical issue. However, in the course of cognitive inquiry, we can concretize the concept of life through metaphors, thereby deepening our comprehension of it. For instance, people commonly understand life through metaphors such as “a journey” and “a dream”. Such expressions can be defined as life metaphors, cognitive tools that reveal the nature, meaning, or characteristics of life. As noted by Lakoff (1992), a prominent scholar in cognitive linguistics, the essence of metaphor lies in a cognitive process whereby abstract experiences (the target domain) are conceptualized through concrete ones (the source domain). At its core, metaphor involves the mapping of properties from the source domain to the target domain, thereby generating a systematic set of conceptual correspondences.

Recently, researchers have begun exploring the relationship between life metaphors and meaning in life. Bochaver and Fenko (2010) found that the public figures preferred “life is a journey”, while the help-seekers frequently used “life as death”. Life metaphors have also been shown to influence students’ motivation and choices (Hoffman et al., 2015). Notably, American adults who adopt the “life is a journey” metaphor are associated with higher meaning in life than those who do not (Baldwin et al., 2017). Landau (2018) argued that life metaphors function as cognitive strategies. Metaphors help individuals perceive life’s continuity, purpose, and value. From the perspective of narrative identity theory, metaphors offer a coherent cognitive schema that helps individuals organize disparate experiences into a unified self-narrative (McAdams & McLean, 2013). For example, the metaphor “life is a journey” shapes an individual’s representation of goals. It also influences their interpretive style when facing setbacks, leading them to perceive obstacles as temporary rather than terminal failures. Cognitive consistency theory similarly suggests that when individuals adopt a life metaphor, they tend to selectively attend to and interpret events in ways that conform to that metaphorical framework. This process improves perceived coherence, purpose, and significance—the three common themes of meaning in life (Heintzelman & King, 2014). Thus, life metaphors are not only linguistic expressions. They are also cognitive tools that determine how meaning is constructed. Yet, the relationship between life metaphors and meaning in life may be affected by cultural context, individual differences, and other psychological variables. In the Chinese sociocultural context, the association between life metaphors and meaning in life is still underexplored.

Prior research shows that depression significantly undermines meaning in life (Schulenberg et al., 2016; Jing & Ding, 2024; Kim & Ko, 2025). However, the extent to which life metaphors serve as cognitive resources for meaning-making may not be uniform across all individuals. This relationship is likely contingent upon depressive symptoms. Depressive symptoms are characterized by the negative cognitive triad: pervasive negative beliefs about the self, world, and future (Beck & Bredemeier, 2016). Individuals with depressive symptoms selectively attend to negative information (Peckham et al., 2010) and ignore positive information (Winer & Salem, 2016). Such information-processing biases may prevent individuals from effectively using life metaphors. Thus, the present study intends to explore whether depressive symptoms moderate the relationship between life metaphors and meaning in life, such that this association may be weaker at higher levels of depressive symptoms. Hence, this study advances our knowledge of the boundary conditions under which life metaphors influence meaning in life.

In summary, prior evidence indicates that life metaphors are closely related to individuals’ meaning in life; however, research in this area has been hindered by the lack of a validated instrument among Chinese college students. Crego et al. (2022) developed the 19-item Life Metaphor Questionnaire (LMQ), which has shown sound psychometric properties in validation studies. Yet, its fitness for Chinese college students has not been tested. Moreover, the interplay between life metaphors, depressive symptoms, and life meaning remains unclear. Accordingly, this study consists of two parts. Study 1 aims to revise and validate the Life Metaphor Questionnaire among Chinese college students. Study 2 seeks to empirically examine the relationship among life metaphors, depressive symptoms, and meaning in life. Overall, this study will test the hypothesis that depressive symptoms moderate the association between life metaphors and meaning in life, such that this association may be weaker at higher levels of depressive symptoms. Specifically, this study contributes to the literature in two ways. First, from a methodological perspective, it provides an instrument for assessing life metaphors among Chinese college students. Second, from a theoretical perspective, the present study integrates cognitive linguistics with clinical and developmental psychology by exploring whether depressive symptoms moderate the relationship between life metaphors and meaning in life.

## 2. Study 1: Revision of the Life Metaphor Questionnaire Among Chinese College Students

### 2.1. Method

#### 2.1.1. Participants

A convenience sampling method was used to recruit participants from two universities in Guangdong Province, China. Group-administered online surveys conducted in classroom settings on Wenjuanxing (http://www.wjx.cn) yielded a final sample of 1996 students after excluding incomplete or invalid questionnaires. The sample was randomly split into two groups: Subsample 1 (877 valid responses; 301 males, 576 females; *M* = 18.89, *SD* = 1.34) was designated for item analysis and EFA, while Subsample 2 (1119 valid responses; 283 males, 836 females; *M* = 18.95, *SD* = 1.04) was used for CFA and reliability assessments. The study received Ethics Committee approval (protocol number: 2023-421) from South China Normal University and all participants gave informed consent.

#### 2.1.2. Procedures

We validated the original English version of the Life Metaphor Questionnaire (Crego et al., 2022) through rigorous translation procedures. First, Translation and Back-translation of the questionnaire. With the original authors’ permission, two independent translators—a doctoral-level psychologist and a master’s-level psychology researcher—translated the 19-item questionnaire from English to Chinese. Discrepancies and ambiguous items were discussed and resolved to produce a preliminary Chinese version. This version was then back-translated into English by a third bilingual researcher, and the back-translated version was compared with the original to ensure semantic equivalence and accuracy. Second, cross-cultural adjustment of the questionnaire. The Chinese adaptation of the LMQ was developed through cultural adaptation evaluation and content modification by a team of psychology professors and graduate students. Third, Item Evaluation and Refinement. A total of 45 valid responses were collected from Chinese college participants to evaluate and refine the translated questionnaire items. Participants were instructed to identify and provide rationales for any items they found unclear, using a structured prompt: “Please mark items you do not understand and explain the reasons for your confusion.” For items flagged as ambiguous, revisions were made based on participants’ feedback and expert re-deliberations, with consideration of cultural adaptability. The final preliminary Chinese version of the LMQ consisted of 19 items rated on a 7-point Likert questionnaire, ranging from 1 (“strongly disagree”) to 7 (“strongly agree”). Fourth, Formal Investigation. A total of 1996 valid responses were collected from Chinese college students to examine the questionnaire’s structural properties. A formal questionnaire was developed after screening out unsuitable items using item analysis, EFA, CFA, and reliability analysis.

#### 2.1.3. Statistical Analysis

In Study 1, we conducted all analyses in two stages using two independent subsamples. Item analysis and EFA were performed on Subsample 1 (*n* = 877) in SPSS 26.0 within the framework of Classical Test Theory (CTT). Before the item correlation analysis, the normality of all variables was examined using the Shapiro–Wilk test. The results indicated that the data did not follow a normal distribution. Therefore, Spearman’s correlation coefficients were computed to examine correlations between each item and the total score among the LMQ items. For item analysis, items were also screened using independent-samples T-tests. A critical ratio reaching statistical significance (*p* < 0.05) was used as the criterion for adequate item discrimination.

The factor structure identified in the EFA was validated on Subsample 2 (*n* = 1119) using CFA in Mplus 8.3. Fit indices were assessed according to Hu and Bentler (1999). The following fit indices were employed to assess the goodness of fit: the Comparative Fit Index (good fit is characterized by a score greater than 0.90 and excellent fit exceeds 0.95), the Tucker–Lewis Index (good fit is characterized by a score greater than 0.90 and excellent fit exceeds 0.95), the Standardized Root Mean Square Residual (values under 0.08 indicate adequate fit and scores below 0.05 are considered good fit), and the Root Mean Square Error of Approximation (scores under 0.06 are considered excellent fit and values under 0.08 are considered adequate fit). Reliability analysis was conducted in Subsample 2 using Cronbach’s alpha (computed in SPSS 26.0) and McDonald’s omega. Omega coefficients were derived from the standardized loadings of the CFA solution in Mplus 8.3. The EFA explored the preliminary factor structure, and the CFA in an independent sample validated the model.

### 2.2. Results

#### 2.2.1. Item Analysis

Item analysis was conducted on Subsample 1 (*n* = 877) using the critical ratio (CR) and item–total correlation as indices of item identification. Using the extreme-group method, participants were ranked by total scores, with the bottom 27% (*n* = 237) as the low group and the top 27% (*n* = 237) as the high group. Independent-samples T-tests compared scores between these groups for each item. All 19 items showed significant differences (all *p* < 0.01), demonstrating adequate discriminative power. Spearman correlation coefficients between each item and the total score were computed, with a coefficient greater than 0.30 adopted as the retention criterion. The Spearman correlation coefficients between each item and the total score ranged from 0.325 to 0.759, all of which were statistically significant (*p* < 0.001). Since every item met the criteria for discrimination, all 19 items were retained for EFA.

#### 2.2.2. Exploratory Factor Analysis

**Figure 1:**
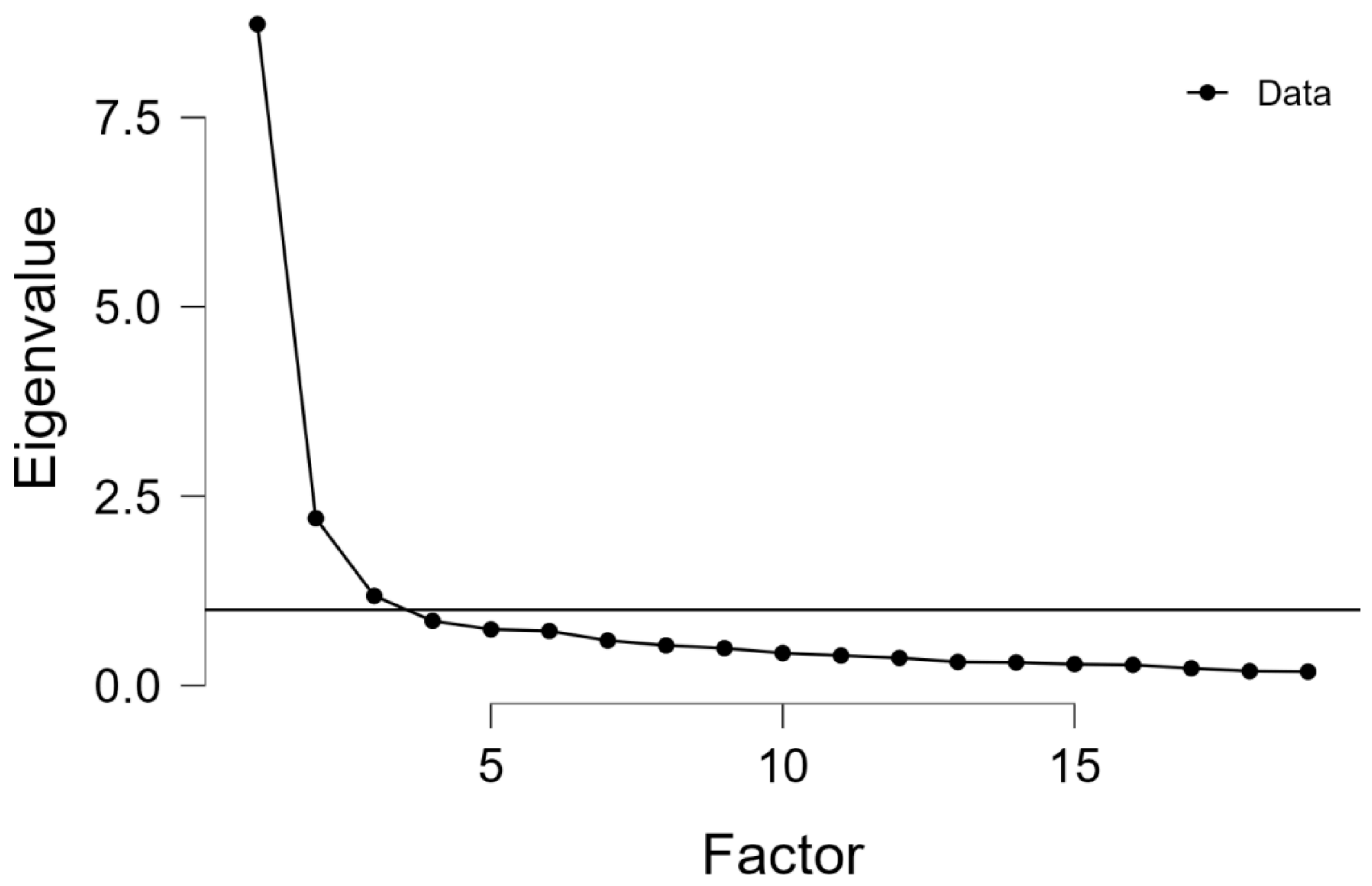
Scree plot of the exploratory factor analysis. The horizontal line represents an eigenvalue of 1 (Kaiser criterion); factors with eigenvalues above this line are retained.

EFA was conducted on Subsample 1 (*n* = 877). The Kaiser–Meyer–Olkin (KMO) measure of sampling adequacy was 0.931, and Bartlett’s test of sphericity was significant (*χ*^2^ = 10,518.065, *df* = 171, *p* < 0.001), indicating that the data were suitable for EFA. The principal component analysis with varimax rotation was employed to extract factors. Based on eigenvalues greater than 1 and a scree plot inspection, a three-factor solution was retained (see Figure 1). Six items with cross-loadings on multiple factors (Items 3, 6, 7, 12, 17, and 18) were removed. Combining the theoretical structure of the original LMQ, the final 13-item questionnaire was organized into three dimensions: fiction-related metaphors (e.g., “life is a sports game”), uncertainty metaphors (e.g., “life is an adventure”), and eudaimonic metaphors (e.g., “life is a journey”) (see Table 1). All retained items showed high factor loadings, ranging from 0.621 to 0.882. The three factors collectively explained 68.08% of the total variance, indicating strong structural validity.

#### 2.2.3. Confirmatory Factor Analysis

We first conducted a baseline CFA of the original 19-item three-factor model in Sample 2. The model fit was inadequate: *χ*^2^/*df* = 22.248, TLI = 0.736, CFI = 0.770, SRMR = 0.092, RMSEA = 0.138. Next, we tested the 13-item model structure derived from EFA. The indices of the 13-item model fit indicated an acceptable fit. However, item LM19 (“life is a prison”) showed a relatively low factor loading (0.456) on the factor of uncertainty metaphors, which was notably weaker than other items (*M* = 0.797, *SD* = 0.078). Moreover, the R^2^ of LM19 was the lowest among all observed variables (0.208), indicating that the latent factor accounted for only 20.8% of its total variance. We therefore tested a 12-item model excluding LM19. The fit indices for the 12-item model were as follows: *χ*^2^/*df* = 6.845, TLI = 0.952, CFI = 0.963, SRMR = 0.037, RMSEA = 0.072. All indices exceeded acceptable thresholds, with most indicating an excellent fit. Therefore, the 12-item model was selected as the final structure of the LMQ for Chinese college students, comprising 12 items across three dimensions: fiction-related metaphors, uncertainty metaphors, and eudaimonic metaphors (see Table 2, Appendix A).

#### 2.2.4. Reliability Analysis

Reliability analysis was conducted on Subsample 2 using both Cronbach’s alpha and McDonald’s omega. For the overall 12-item Life Metaphor Questionnaire, α = 0.912, ω = 0.912, indicating excellent internal consistency. For the subscales, eudaimonic metaphors (α = 0.882, ω = 0.883), fiction-related metaphors (α = 0.873, ω = 0.875), and uncertainty metaphors (α = 0.871, ω = 0.876), thus demonstrating good internal consistency.

### 2.3. Discussion

Study 1 aimed to revise the Chinese version of LMQ to provide a rigorous instrument for investigating life metaphors among college students. The EFA demonstrated that a three-factor structure accounted for 68.08% of the total variance. The Chinese version of LMQ retained three dimensions consistent with the original English version (Crego et al., 2022): fiction-related metaphors, uncertainty metaphors, and eudaimonic metaphors. Subsequent CFA further supported this three-factor model, demonstrating acceptable model fit (TLI = 0.952, CFI = 0.963, SRMR = 0.037, RMSEA = 0.072). In addition, the Chinese version of LMQ demonstrated excellent internal consistency. For the overall 12-item questionnaire, α = 0.912, ω = 0.912. The subscales also showed good reliability: eudaimonic metaphors (α = 0.882, ω = 0.883), fiction-related metaphors (α = 0.873, ω = 0.875), and uncertainty metaphors (α = 0.871, ω = 0.876). Collectively, these psychometric findings indicate that the 12-item questionnaire is a reliable measure with a three-factor structure for assessing life metaphors among Chinese college students. Study 2 aims to examine whether life metaphors relate to meaning in life and whether depressive symptoms moderate this association among Chinese college students.

## 3. Study 2: Depressive Symptoms as a Moderator in the Relationship Between Life Metaphor and Meaning in Life

### 3.1. Method

#### 3.1.1. Participants

A convenience sampling method was employed to recruit participants from two universities in Guangdong Province, China. Group-administered online surveys were conducted in classroom settings using Wenjuanxing (http://www.wjx.cn), resulting in 746 valid responses (387 males, 359 females; *M* = 18.68, *SD* = 1.20). The study received Ethics Committee approval (protocol number: 2023-421) from South China Normal University and all participants gave informed consent.

#### 3.1.2. Instruments

The Life Metaphor Questionnaire (LMQ) includes three subscales: fiction-related metaphors, uncertainty metaphors, and eudaimonic metaphors, comprising 12 items in total. Responses were rated on a 7-point Likert scale ranging from 1 (“strongly disagree”) to 7 (“strongly agree”), with the total LMQ score ranging from 12 to 84. Higher scores indicate a stronger general endorsement of life metaphors or a greater tendency to understand life through metaphorical thinking. The Cronbach’s alpha of LMQ in the present study was 0.885, and the McDonald’s omega was 0.886.

The Meaning in Life Questionnaire (MLQ), originally developed by Steger et al. (2006), contains 10 items and uses a 7-point Likert scale ranging from 1 (“not at all true”) to 7 (“completely true”). It assesses the presence of life (e.g., “I understand my life’s meaning”) and the search for meaning in life (e.g., “I am always looking to find my life’s purpose”), with the total MLQ score ranging from 10 to 70. Higher scores reflect a higher level of meaning in life. The Chinese version of the MLQ has been widely used and demonstrates good reliability and validity (Luo et al., 2022). The Cronbach’s alpha of the current sample was 0.840, and the McDonald’s omega was 0.812.

The Patient Health Questionnaire-9 (PHQ-9) was developed by Spitzer et al. (1999). PHQ-9 is a widely used and validated self-report instrument in mental health research, particularly in primary care and epidemiological studies. It contains 9 items to assess the presence and severity of depressive symptoms based on the DSM-IV criteria for major depressive disorder (e.g., “Little interest or pleasure in doing things”). Responses were rated on a 4-point Likert scale ranging from 0 (“not at all”) to 3 (“nearly every day”), with the total PHQ-9 score ranging from 0 to 27. A PHQ-9 total score of 10 or higher may suggest the presence of depressive symptoms. Higher scores indicate more severe depressive disorder. The Chinese version of the PHQ-9 has been widely used and demonstrates good reliability and validity (Zhang et al., 2013). In this study, the Cronbach’s alpha of the current sample was 0.871, and the McDonald’s omega was 0.873.

#### 3.1.3. Statistical Analysis

Statistical analyses were performed using SPSS 26.0, the SPSS PROCESS macro (Hayes, 2022), and Mplus 8.3. (1) Before the item correlation analysis, the normality of all variables was examined using the Shapiro–Wilk test. The results indicated that the data did not follow a normal distribution. Therefore, Spearman’s correlation coefficients were computed to examine correlations among variables, including life metaphors, depressive symptoms, and meaning in life, using SPSS 26.0. Correlation coefficients were interpreted following the guidelines proposed by Gignac and Szodorai (2016), with values of 0.10, 0.20, and 0.30 considered small, typical, and relatively large, respectively. (2) Reliability analysis was conducted using Cronbach’s alpha (computed in SPSS 26.0) and McDonald’s omega (computed in Mplus 8.3). (3) Moderated regression analyses were conducted using the SPSS PROCESS macro (Model 1) to examine whether depressive symptoms moderated the associations between the three dimensions of life metaphors (fiction-related metaphors, uncertainty metaphors, eudaimonic metaphors) and meaning in life. All continuous variables were standardized. A bootstrapping procedure with 5000 resamples was used to simultaneously estimate the direct and indirect effects and obtain their 95% confidence intervals (95% CI).

### 3.2. Results

#### 3.2.1. Common Method Bias Assessment

Harman’s single-factor test was used to evaluate common method bias. An EFA without rotation included all items from life metaphors, depressive symptoms, and meaning in life. Six factors had eigenvalues greater than 1, with the first factor explaining 24.15% of the total variance—well below the 40% threshold. Thus, common method bias was not a significant issue in this study.

#### 3.2.2. Descriptive Statistics and Correlation Matrix

The Spearman correlation results are presented in Table 3 below. Fiction-related metaphors, uncertainty metaphors, eudaimonic metaphors, and meaning in life were significantly positively correlated with each other. Depressive symptoms were significantly negatively correlated with fiction-related metaphors (*ρ* = −0.170, *p* < 0.001), eudaimonic metaphors (*ρ* = −0.272, *p* < 0.001), and meaning in life (*ρ* = −0.327, *p* < 0.001). But depressive symptoms showed a very weak positive association with uncertainty metaphors (*ρ* = 0.077, *p* < 0.05). At the facet level, depressive symptoms showed a relatively large correlation with the presence of meaning (*ρ* = −0.420, *p* < 0.001), whereas their correlation with search for meaning was small (*ρ* = −0.137, *p* < 0.001), following Gignac and Szodorai’s (2016) guidelines.

#### 3.2.3. Moderating Effect of Depressive Symptoms on the Relationship Between Eudaimonic Metaphors and Meaning in Life

We tested whether depressive symptoms moderated the relationship between eudaimonic metaphors and meaning in life, controlling for gender and age (see Table 4). The MLQ comprises two distinct dimensions, presence of meaning and search for meaning; we examined these two dimensions separately.

**Figure 2:**
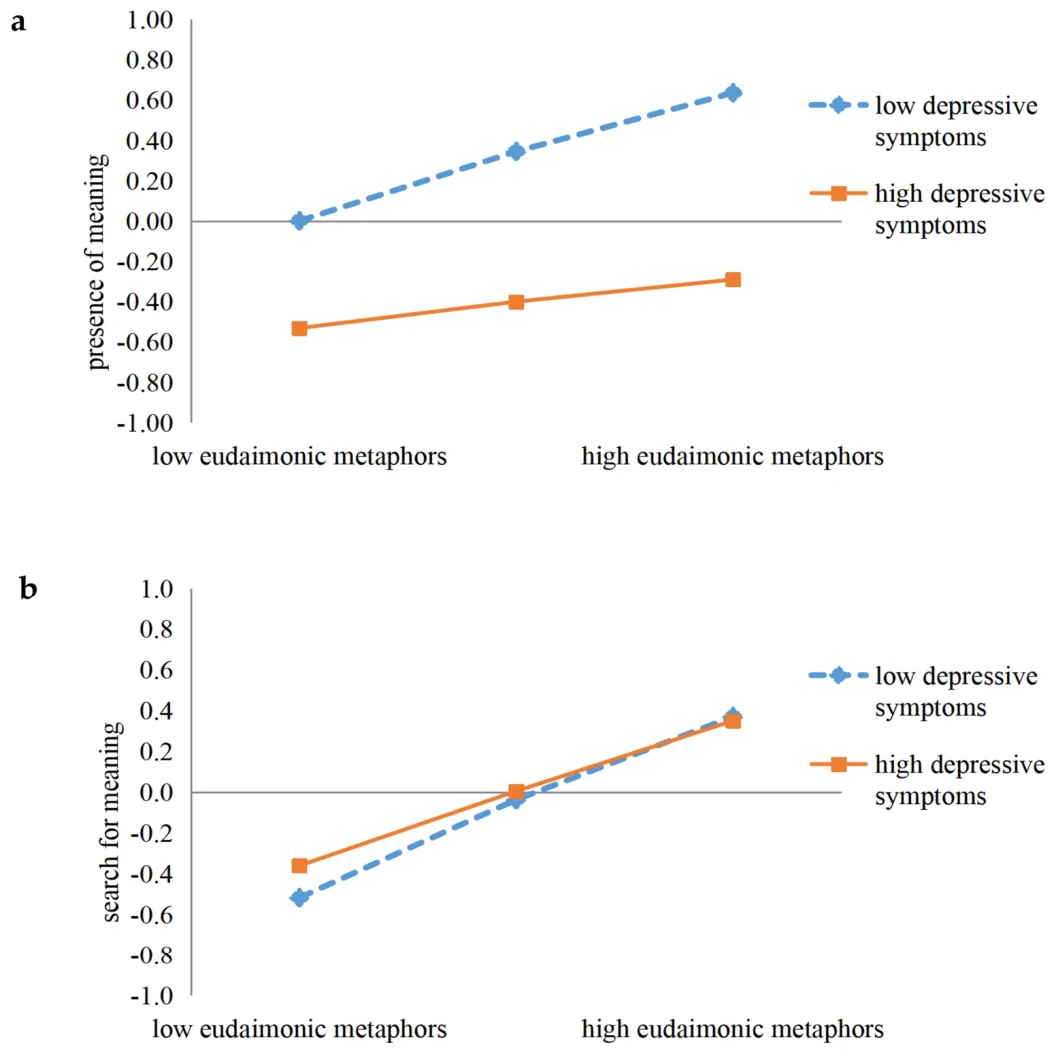
Interaction between eudaimonic metaphors and depressive symptoms on meaning in life. (**a**) denotes presence of meaning as the outcome variable and (**b**) denotes search for meaning as the outcome variable.

When depressive symptoms were included in the model, the interaction of eudaimonic metaphors and depressive symptoms was significantly associated with presence of meaning (*β* = −0.107, *t* = −4.247, *p* < 0.001, 95% CI [−0.156, −0.057]). Adding the interaction term significantly improved model fit, Δ*R*^2^ = 0.019, Δ*F*(1, 740) = 18.041, *p* < 0.001. The final model explained 23.1% of the variance (*R*^2^ = 0.231). The results indicated that depressive symptoms significantly moderated the associations between eudaimonic metaphors and presence of meaning. To further interpret this interaction, simple slopes analyses examined the effect of depressive symptoms on presence of meaning at high (+1*SD*) and low (−1*SD*) levels (see Table 4 and Figure 2a). The results showed that, at low levels of depressive symptoms (−1*SD*), eudaimonic metaphors were significantly and positively associated with the presence of meaning (*β* = 0.344, *t* = 7.369, *p* < 0.001, 95% CI [0.252, 0.436]). Similarly, under high levels of depressive symptoms (+1*SD*), eudaimonic metaphors were still significantly and positively associated with the presence of meaning, though to a lesser extent (*β* = 0.130, *t* = 3.455, *p* = 0.001, 95% CI [0.056, 0.205]). These findings indicated that the positive association between eudaimonic metaphors and presence of meaning was stronger at lower levels of depressive symptoms, supporting a significant moderating role of depressive symptoms.

Similarly, the interaction of eudaimonic metaphors and depressive symptoms was significantly associated with search for meaning (*β* = −0.058, *t* = −2.215, *p* < 0.05, 95% CI [−0.109, −0.007]). Adding the interaction term significantly improved model fit, Δ*R*^2^ = 0.006, Δ*F*(1, 740) = 4.904, *p* < 0.05. The final model explained 17.0% of the variance (*R*^2^ = 0.170). The results indicated that depressive symptoms significantly moderated the associations between eudaimonic metaphors and search for meaning. To interpret this interaction further, simple slopes analyses examined the effect of depressive symptoms on search for meaning at high (+1*SD*) and low (−1*SD*) levels (see Table 4 and Figure 2b). The results showed that, at low levels of depressive symptoms (−1*SD*), eudaimonic metaphors were significantly and positively associated with the search for meaning (*β* = 0.482, *t* = 9.926, *p* < 0.001, 95% CI [0.386, 0.577]). Similarly, under high levels of depressive symptoms (+1*SD*), eudaimonic metaphors were still significantly and positively associated with search for meaning, though to a lesser extent (*β* = 0.366, *t* = 9.324, *p* < 0.001, 95% CI [0.289, 0.443]). These findings indicated that the positive association between eudaimonic metaphors and search for meaning was stronger at lower levels of depressive symptoms, supporting a significant moderating role of depressive symptoms.

#### 3.2.4. Moderating Effect of Depressive Symptoms on the Relationship Between Fiction-Related Metaphors and Meaning in Life

Following the same analytical strategy, we tested whether depressive symptoms moderated the relationship between fiction-related metaphors and meaning in life, controlling for gender and age (see Table 5). We also examined presence of meaning and search for meaning separately.

**Figure 3:**
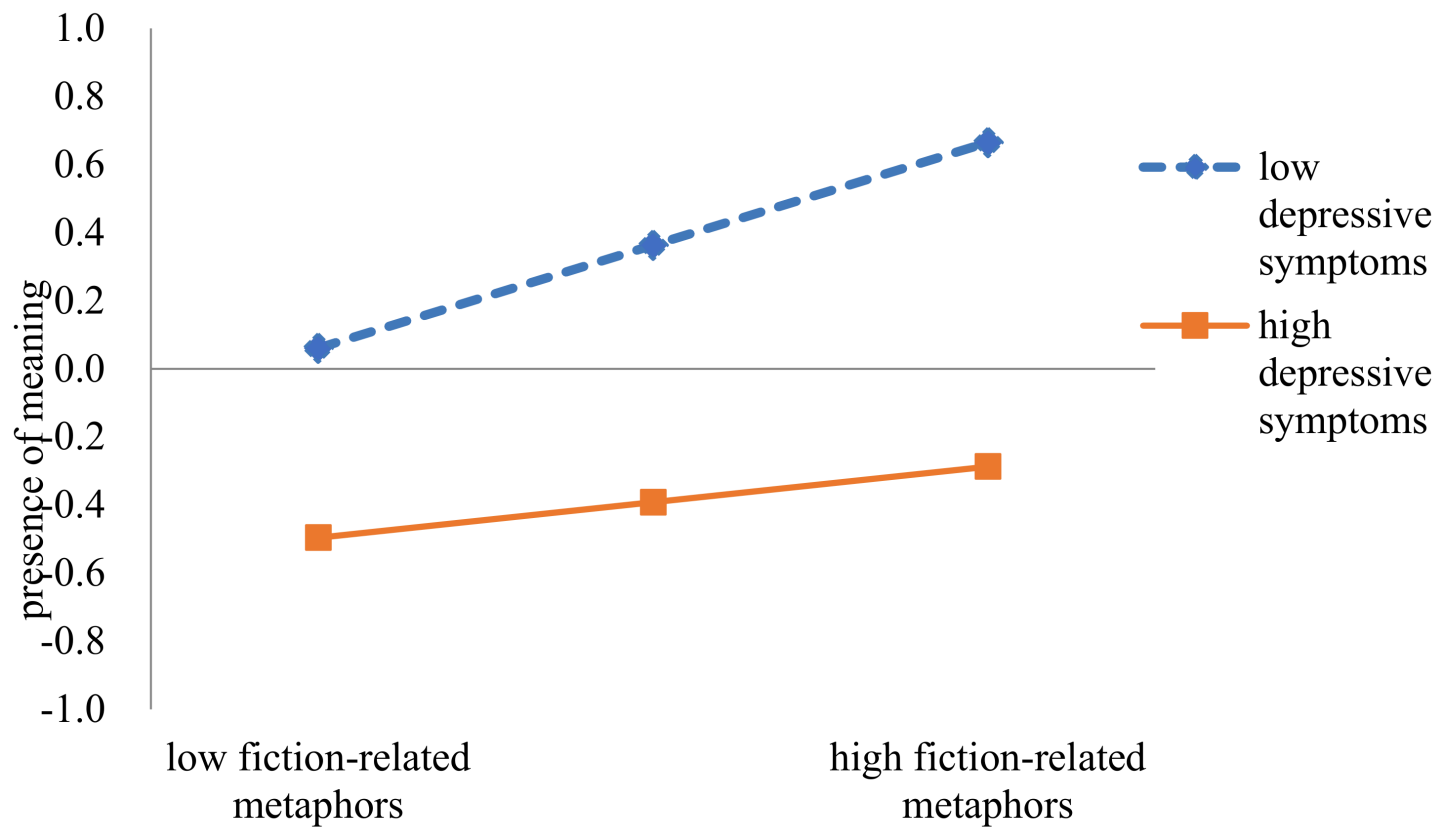
Interaction between fiction-related metaphors and depressive symptoms on presence of meaning.

The results indicated that depressive symptoms significantly moderated the associations between fiction-related metaphors and presence of meaning. When depressive symptoms were included in the model, the interaction of fiction-related metaphors and depressive symptoms was significantly associated with presence of meaning (*β* = −0.099, *t* = −3.188, *p* = 0.001, 95% CI [−0.160, −0.038]). Adding the interaction term significantly improved model fit, Δ*R*^2^ = 0.011, Δ*F*(1, 740) = 10.165, *p* = 0.001. The final model explained 22.4% of the variance (*R*^2^ = 0.224). To further interpret this interaction, simple slopes analyses examined the effect of depressive symptoms on presence of meaning at high (+1*SD*) and low (−1*SD*) levels (see Table 5 and Figure 3). The results showed that, at low levels of depressive symptoms (−1*SD*), fiction-related metaphors were significantly and positively associated with the presence of meaning (*β* = 0.302, *t* = 6.764, *p* < 0.001, 95% CI [0.214, 0.390]). Similarly, under high levels of depressive symptoms (+1*SD*), fiction-related metaphors were still significantly and positively associated with the presence of meaning, though to a lesser extent (*β* = 0.104, *t* = 2.290, *p* < 0.05, 95% CI [0.015, 0.194]). These findings indicated that the positive association between fiction-related metaphors and presence of meaning was stronger at lower levels of depressive symptoms, supporting a significant moderating role of depressive symptoms.

However, depressive symptoms did not moderate the associations between fiction-related metaphors and search for meaning. The interaction of fiction-related metaphors and depressive symptoms was not significantly associated with search for meaning (*β* = −0.042, *t* = −1.258, *p* > 0.05, 95% CI [−0.107, 0.024]). Adding the interaction term did not significantly improve the model, Δ*R*^2^ = 0.002, *F*(1, 740) = 1.583, *p >* 0.05. The final model explained 9.9% of the variance (*R*^2^ = 0.099).

#### 3.2.5. Moderating Effect of Depressive Symptoms on the Relationship Between Uncertainty Metaphors and Meaning in Life

We finally tested whether depressive symptoms moderated the relationship between uncertainty metaphors and meaning in life, controlling for gender and age (see Table 6). We also examined presence of meaning and search for meaning separately. The results indicated that depressive symptoms did not moderate the associations between uncertainty metaphors and presence of meaning. The interaction of uncertainty metaphors and depressive symptoms was not significantly associated with presence of meaning (*β* = −0.002, *t* = −0.076, *p >* 0.05, 95% CI [−0.063, 0.059]). Adding the interaction term did not significantly improve the model, Δ*R*^2^ = 0, *F*(1, 740) = 0.006, *p >* 0.05. The final model explained 17.3% of the variance (*R*^2^ = 0.173).

Moreover, the results also indicated that depressive symptoms did not moderate the associations between uncertainty metaphors and search for meaning. The interaction of uncertainty metaphors and depressive symptoms was not significantly associated with search for meaning (*β* = 0.022, *t* = 0.663, *p >* 0.05, 95% CI [−0.043, 0.087]). Adding the interaction term did not significantly improve the model, Δ*R*^2^ = 0.001, *F*(1, 740) = 0.439, *p >* 0.05. The final model explained 6.6% of the variance (*R*^2^ = 0.066).

### 3.3. Discussion

Study 2 examined the relationship among life metaphors, depressive symptoms, and meaning in life. Fiction-related metaphors, uncertainty metaphors, eudaimonic metaphors, and meaning in life were significantly and positively correlated with each other. Eudaimonic metaphors showed the strongest correlation with meaning in life (*ρ* = 0.439, *p* < 0.001), followed by fiction-related metaphors (*ρ* = 0.341, *p* < 0.001), whereas uncertainty metaphors showed only a small correlation (*ρ* = 0.095, *p* < 0.001). Depressive symptoms were significantly negatively correlated with fiction-related metaphors (*ρ* = −0.170, *p* < 0.001), eudaimonic metaphors (*ρ* = −0.272, *p* < 0.001), and meaning in life (*ρ* = −0.327, *p* < 0.001). But depressive symptoms showed a very weak positive association with uncertainty metaphors (*ρ* = 0.077, *p* < 0.05). At the facet level, depressive symptoms showed a relatively large correlation with the presence of meaning (*ρ* = −0.420, *p* < 0.001), whereas their correlation with search for meaning was small (*ρ* = −0.137, *p* < 0.001). A bootstrap test (5000 resamples) confirmed that the former correlation was significantly stronger than the latter, 95% CI [−0.365, −0.205].

The moderating role of depressive symptoms was tested separately for each meaning dimension across three dimensions of life metaphors. Depressive symptoms significantly moderated the associations of eudaimonic metaphors with both the presence of meaning (*β* = −0.107, *t* = −4.247, *p* < 0.001, 95% CI [−0.156, −0.057]) and the search for meaning (*β* = −0.058, *t* = −2.215, *p* < 0.05, 95% CI [−0.109, −0.007]); moderated the association of fiction-related metaphors with the presence of meaning (*β* = −0.099, *t* = −3.188, *p* = 0.001, 95% CI [−0.160, −0.038]), but not with the search for meaning (*β* = −0.042, *t* = −1.258, *p >* 0.05, 95% CI [−0.107, 0.024]); and did not moderate the associations of uncertainty metaphors with both the presence of meaning (*β* = −0.002, *t* = −0.076, *p >* 0.05, 95% CI [−0.063, 0.059]) and the search for meaning (*β* = 0.022, *t* = 0.663, *p >* 0.05, 95% CI [−0.043, 0.087]).

## 4. General Discussion

### 4.1. Adaptation and Validation of the LMQ

Study 1 initially focused on the adaptation and validation of the LMQ among college students. The EFA demonstrated that the Chinese version of the LMQ retained three dimensions consistent with the original English version (Crego et al., 2022): fiction-related metaphors, uncertainty metaphors, and eudaimonic metaphors. Crego et al. treated fiction metaphors as a hybrid category within eudaimonic and uncertainty. But in the present Chinese college sample, fiction metaphors emerged as an independent factor. This divergence may reflect cultural and population differences. Fiction-related metaphors (e.g., life is a school or a teacher, a sports competition, or a stage play) framing life as structured, rule-governed social scenarios may be more distinct in a collectivistic cultural context. The CFA supported the three-factor structure and the overall model, showing acceptable fit. The findings indicated that the 12-item questionnaire possessed satisfactory psychometric properties. In Study 2, the three dimensions of the LMQ were significantly and positively correlated with the total score of meaning in life, providing preliminary evidence for the scale’s convergent validity. These findings establish the LMQ as a reliable tool for assessing life metaphors in Chinese-speaking populations.

### 4.2. Life Metaphors and Meaning in Life

Study 2 showed that fiction-related metaphors, uncertainty metaphors, eudaimonic metaphors, and meaning in life were positively correlated with each other. This result was consistent with prior research showing that meaning in life can be framed through accessible metaphors (Baldwin et al., 2017). Metaphor is not simply a linguistic device but a cognitive tool through which individuals understand abstract and complicated ideas in terms of more concrete, structured concepts (Landau, 2018; Kövecses, 2015). For college students, life metaphors provide cognitive schemas that transform abstract concepts into understandable frameworks. As a result, life metaphors were linked to the construction of meaning in life. For example, the metaphor of “life as a journey” helps students attend to the scenery along the way. The journey metaphor emphasizes the value of each life experience rather than focusing solely on outcomes.

Interestingly, our results showed that uncertainty metaphors were positively correlated with meaning in life among Chinese college students, which was inconsistent with prior results. Crego et al. (2022) investigated people in Spain and Latin America and found that uncertainty metaphors were negatively associated with feeling meaning in life. One possible explanation is that, in the context of Chinese culture, the interpretation of the uncertainty metaphor might differ from that in Spain and Latin America. Within a cultural framework that values dialectical thinking and sees opposites as coexisting, the uncertainty metaphor (e.g., “life is a maze”) may be perceived not solely as threatening but as an integral part of life in the Chinese context. We suggest that, within a cultural tradition of dialectical thinking that construes opposites as coexisting (Peng & Nisbett, 1999), acknowledging life’s uncertainty is not experienced as a threat to felt meaning. Instead, it reflects an open, exploratory orientation toward life’s complexity, conceptually close to the curiosity and openness that characterize adaptive search for meaning (Steger et al., 2008). This interpretation is consistent with our finding that uncertainty metaphors were unrelated to the presence of meaning but positively associated with the search for meaning among Chinese college students.

### 4.3. Depressive Symptoms as a Moderator

Depressive symptoms showed a relatively large correlation with the presence of meaning, whereas their correlation with the search for meaning was small, consistent with recent meta-analytic evidence (He et al., 2023; Ouyang et al., 2026). A structural account of this asymmetry is that the presence of meaning emphasizes cognition, focusing on individuals’ perception of life meaning. The search for meaning highlights motivation, centering on individuals’ pursuit of life meaning (He et al., 2023). Depressive symptoms centrally involve hopelessness, anhedonia, and a lessened sense of mattering, all of which directly undermine the cognitive framework of felt meaning. The search for meaning, by contrast, is a motivational orientation whose adaptive value depends on whether the search is ultimately satisfied. The association between the search for meaning and depressive symptoms is correspondingly weak and unstable (He et al., 2023; Steger et al., 2008). This asymmetric coupling may account for the moderating pattern observed in the present study. Because depressive symptoms are strongly linked to the presence of meaning but only loosely coupled with the search for meaning, they have far greater leverage to condition the association between life metaphor and the presence of meaning than between life metaphor and the search for meaning.

A clear gradient emerged in the moderating role of depressive symptoms across the three metaphor dimensions and the two facets of meaning in life: significant moderation effects were consistently stronger among students with low depressive symptoms than among those with high depressive symptoms, and their magnitude decreased systematically from eudaimonic to fiction-related to uncertainty metaphors. Specifically, depressive symptoms significantly moderated the associations of eudaimonic metaphors with both the presence of and the search for meaning; moderated the association of fiction-related metaphors with the presence of meaning, but not with the search for meaning; and did not moderate the associations of uncertainty metaphors with both the presence of and the search for meaning. This graded pattern may reflect the distinct psychological functions of the three metaphor dimensions. Prior research shows that metaphors are not merely linguistic expressions but are cognitive tools that determine how meaning is constructed (Huang & Aaker, 2019; Landau, 2018). Eudaimonic metaphors (e.g., life as a journey, a book or a movie) frame life as an emerging narrative endowed with direction, plot, and intrinsic value. Fiction-related metaphors (e.g., life as a school or a teacher, a sports competition, or a stage play) frame life as structured, rule-governed social situations. Uncertainty metaphors (e.g., life as a lottery, a game of chance, a riddle, or a puzzle) frame life as uncontrollable and challenging. Eudaimonic metaphors showed the strongest correlation with meaning in life, followed by fiction-related metaphors, whereas uncertainty metaphors showed only a small correlation. Individuals with elevated depressive symptoms exhibit biases in information processing, particularly a reduced ability to utilize positive information (Peckham et al., 2010). Moreover, individuals with depressive symptoms often experience negative self-evaluation (Beck & Bredemeier, 2016). They either ignore or reinterpret the positive semantics of the life metaphor through a negative interpretative lens. In contrast, students with low depressive symptoms are more likely to accept these metaphors, linking metaphorical frameworks to self-narratives associated with greater meaning in life. Because the meaning-ladenness of the metaphors weakens from eudaimonic to fiction-related and uncertainty metaphors, there is progressively less for depressive biases to act upon, which may explain why the moderating effect fades along the same gradient.

## 5. Practical Implications

The present findings have important implications for research and psychological health practice among college students. First, this research provides a reliable instrument for assessing life metaphors, enabling future cross-cultural investigations. Second, the distinct three-factor structure of the LMQ highlights the multidimensional nature of life metaphors among Chinese college students. Third, the present study highlights the important role of depressive symptoms in the relationship between life metaphors and meaning in life. More broadly, these findings illustrate that everyday linguistic devices are subtly yet meaningfully linked with psychological functioning. These findings suggest that such subtle linguistic signals may serve as accessible windows into meaning-related processes and as potential targets for future longitudinal and experimental research.

## 6. Limitations

The present study has validated the Chinese version of LMQ and identified depressive symptoms as a significant moderator of the relationship between fiction-related and eudaimonic metaphors and meaning in life. It has several limitations that future studies should address. First, the final 12-item model was modified and evaluated within the same CFA subsample, which was a post-hoc refinement. Although the current findings suggest that the 12-item questionnaire has satisfactory psychometric properties in a Chinese college student sample, the structure should be treated as preliminary and should be independently cross-validated in future samples. Second, it used a cross-sectional design, making causal conclusions impossible to verify. Whether life metaphors shape, reflect, or enhance meaning in life remains a question for future longitudinal research. Third, the present study did not directly measure dialectical thinking or other cultural mechanisms, so the proposed account of the uncertainty metaphor is a possible explanation rather than a demonstrated mechanism. Future studies should include more diverse samples from different cultural backgrounds to investigate the life metaphors. Fourth, the study relied on self-report measures, which are susceptible to response bias. Incorporating qualitative methods, such as the Metaphor Identification Process (Pragglejaz Group, 2007), would provide a more comprehensive understanding of how college students use life metaphors.

## 7. Conclusions

The present study demonstrated that the Chinese version of the Life Metaphor Questionnaire retains a three-factor structure comprising fiction-related metaphors, uncertainty metaphors, and eudaimonic metaphors. CFA supported this structure, and the scale showed excellent internal consistency, indicating that the instrument is acceptable for assessing life metaphors among Chinese college students. All three life metaphor dimensions were positively correlated with meaning in life. Depressive symptoms were negatively correlated with fiction-related metaphors, eudaimonic metaphors, and meaning in life, but showed a very weak positive association with uncertainty metaphors. Depressive symptoms significantly moderated the associations of eudaimonic metaphors with both the presence of and the search for meaning; moderated the association of fiction-related metaphors with the presence of meaning, but not with the search for meaning; and did not moderate the associations of uncertainty metaphors with both the presence of and the search for meaning.

## Figures and Tables

**Table 1:** Exploratory factor analysis of the life metaphor items.

	Factor			Communality
	Fiction-Related Metaphors	Uncertainty Metaphors	Eudaimonic Metaphors	
LM1			0.882	0.821
LM4			0.842	0.776
LM2			0.834	0.789
LM9		0.765		0.718
LM10		0.722		0.721
LM8		0.701		0.583
LM19		0.674		0.560
LM5		0.655		0.530
LM15	0.826			0.771
LM13	0.806			0.746
LM11	0.784			0.678
LM14	0.676			0.654
LM16	0.621			0.504
Eigenvalue	5.949	1.764	1.137	
Variance Explained (%)	45.759	13.572	8.747	
Cumulative Variance Explained (%)	45.759	59.331	68.078	

Note: Items with loadings below 0.30 are not displayed for clarity purposes. LM = life metaphor item.

**Table 2:** Model fit indices for confirmatory factor analysis.

	*χ*^2^/*df*	TLI	CFI	SRMR	RMSEA
19-item model	22.248	0.736	0.770	0.092	0.138
13-item model	7.827	0.936	0.949	0.047	0.078
12-item model	**6.845**	**0.952**	**0.963**	**0.037**	**0.072**

Note: *df* = degrees of freedom; TLI = Tucker–Lewis index; CFI = comparative fit index; SRMR = standardized root mean square; RMSEA = root mean square error of approximation; Values in bold indicate the fit indices of the selected model.

**Table 3:** Descriptive statistics and correlations among variables.

	*M*	*SD*	1	2	3	4	5	6	7	8
1. Total score of LMQ	62.65	11.89	1							
2. Fiction-related metaphors	24.19	6.41	0.901 ***	1						
3. Uncertainty metaphors	20.08	5.00	0.812 ***	0.564 ***	1					
4. Eudaimonic metaphors	18.38	3.11	0.587 ***	0.450 ***	0.305 ***	1				
5. Depressive symptoms	14.33	4.39	−0.127 ***	−0.170 ***	0.077 *	−0.272 ***	1			
6. Total score of MLQ	50.90	8.88	0.306 ***	0.341 ***	0.095 **	0.439 ***	−0.354 ***	1		
7. Presence of meaning	23.24	6.10	0.176 ***	0.256 ***	−0.042	0.333 ***	−0.420 ***	0.817 ***	1	
8. Search for meaning	27.66	5.19	0.363 ***	0.331 ***	0.237 ***	0.427 ***	−0.137 ***	0.746 ***	0.294 ***	1

Note: * *p* < 0.05, ** *p* < 0.01, *** *p* < 0.001. *M* = mean; *SD* = standard deviation; LMQ = Life Metaphor Questionnaire; MLQ = Meaning in Life Questionnaire.

**Table 4:** Moderating effect of depressive symptoms in the relationship between eudaimonic metaphors and meaning in life.

Outcome	Variables	*β*	*t*	*p*	95%CI
Presence of meaning	Gender	−0.044	−1.351	0.177	−0.109, 0.020
	Age	0.044	1.348	0.178	−0.020, 0.108
	Eudaimonic metaphors	0.237	6.934 ***	<0.001	0.170, 0.305
	Depressive symptoms	−0.373	−10.954 ***	<0.001	−0.440, −0.306
	Eudaimonic metaphors × depressive symptoms	−0.107	−4.247 ***	<0.001	−0.156, −0.057
	Low depressive symptoms (*M* − 1*SD*)	0.344	7.369 ***	<0.001	0.252, 0.436
	Medium depressive symptoms (*M*)	0.237	6.934 ***	<0.001	0.170, 0.305
	High depressive symptoms (*M* + 1*SD*)	0.130	3.455 ***	0.001	0.056, 0.205
Search for meaning	Gender	−0.027	−0.788	0.431	−0.094, 0.040
	Age	−0.047	−1.373	0.170	−0.114, 0.020
	Eudaimonic metaphors	0.424	11.918 ***	<0.001	0.354, 0.494
	Depressive symptoms	0.021	0.583	0.560	−0.049, 0.090
	Eudaimonic metaphors × depressive symptoms	−0.058	−2.215 *	<0.05	−0.109, −0.007
	Low depressive symptoms (*M* − 1*SD*)	0.482	9.926 ***	<0.001	0.386, 0.577
	Medium depressive symptoms (*M*)	0.424	11.918 ***	<0.001	0.354, 0.494
	High depressive symptoms (*M* + 1*SD*)	0.366	9.324 ***	<0.001	0.289, 0.443

Note: * *p* < 0.05, *** *p* < 0.001. All continuous variables were standardized. CI = confidence interval; *M* = mean; *SD* = standard deviation.

**Table 5:** Moderating effect of depressive symptoms in the relationship between fiction-related metaphors and meaning in life.

Outcome	Variables	*β*	*t*	*p*	95%CI
Presence of meaning	Gender	−0.046	−1.385	0.166	−0.111, 0.019
	Age	0.028	0.865	0.387	−0.036, 0.093
	Fiction-related metaphors	0.203	6.186 ***	<0.001	0.139, 0.268
	Depressive symptoms	−0.377	−11.387 ***	<0.001	−0.442, −0.312
	Fiction-related metaphors × depressive symptoms	−0.099	−3.188 ***	0.001	−0.160, −0.038
	Low depressive symptoms (*M* − 1*SD*)	0.302	6.764 ***	<0.001	0.214, 0.390
	Medium depressive symptoms (*M*)	0.203	6.186 ***	<0.001	0.139, 0.268
	High depressive symptoms (*M* + 1*SD*)	0.104	2.290 *	<0.05	0.015, 0.194
Search for meaning	Gender	−0.027	−0.746	0.456	−0.096, 0.043
	Age	−0.076	−2.139 *	<0.05	−0.146, −0.006
	Fiction-related metaphors	0.298	8.419 ***	<0.001	0.229, 0.368
	Depressive symptoms	−0.029	−0.798	0.425	−0.099, 0.042
	Fiction-related metaphors × depressive symptoms	−0.042	−1.258	0.209	−0.107, 0.024

Note: * *p* < 0.05, *** *p* < 0.001. All continuous variables were standardized. CI = confidence interval; *M* = mean; *SD* = standard deviation.

**Table 6:** Moderating effect of depressive symptoms in the relationship between uncertainty metaphors and meaning in life.

Outcome	Variables	*β*	*t*	*p*	95%CI
Presence of meaning	Gender	−0.055	−1.597	0.111	−0.122, 0.013
	Age	0.046	1.340	0.181	−0.021, 0.113
	Uncertainty metaphors	−0.003	−0.102	0.918	−0.069, 0.063
	Depressive symptoms	−0.405	−11.940 ***	<0.001	−0.471, −0.338
	Uncertainty metaphors × depressive symptoms	−0.002	−0.076	0.940	−0.063, 0.059
Search for meaning	Gender	−0.051	−1.415	0.158	−0.123, 0.020
	Age	−0.049	−1.363	0.173	−0.121, 0.022
	Uncertainty metaphors	0.236	6.618 ***	<0.001	0.166, 0.306
	Depressive symptoms	−0.087	−2.410 *	<0.05	−0.158, −0.016
	Uncertainty metaphors × depressive symptoms	0.022	0.663	0.508	−0.043, 0.087

Note: * *p* < 0.05, *** *p* < 0.001. All continuous variables were standardized. CI = confidence interval.

## Data Availability

The data presented in this study are available on request from the corresponding author. The data are not publicly available because of privacy restrictions under the institutional data protection policy and the informed consent agreements signed by participants. The analysis syntax is available from the corresponding author upon reasonable request.

## Appendix A. The 12-Item Chinese Life Metaphor Questionnaire

Subscale 1: Eudaimonic metaphors

1. Life is a JOURNEY.
  人生是一段旅程。
2. Life is like A BOOK or A MOVIE.
  人生是一本书或一部电影。
3. Life is like A BOX FULL OF EXPERIENCES.
  人生是一个装满经历的盒子。

Subscale 2: Uncertainty metaphors

4. Life is A LOTTERY, A GAME OF CHANCE.
  人生是一张彩票或一场赌博。
5. Life is A WAR or A BATTLE.
  人生是一场战斗或战争。
6. Life is A MAZE.
  人生是一个迷宫。
7. Life is A RIDDLE or A PUZZLE.
  人生是一个谜语或谜题。

Subscale 3: Fiction-related metaphors

8. Life is A SCHOOL or A TEACHER.
  人生是一个学校或一位老师。
9. Life is A SPORTS COMPETITION.
  人生是一场体育比赛。
10. Life is like A STAGE PLAY.
  人生是一场舞台剧。
11. Life is like A HOUSE or A BUILDING.
  人生是一座房子或一座建筑。
12. Life is A CARNIVAL.
  人生是一场狂欢。
